# *Staphylococcus aureus* Adhesion on Hydrophobin Coatings: Adhesion Forces and the Influence of Surface Charge

**DOI:** 10.1021/acsomega.4c11010

**Published:** 2025-08-20

**Authors:** Friederike Nolle, Ben Wieland, Kirstin Kochems, Hannah Heintz, Michael Lienemann, Philipp Jung, Hendrik Hähl, Markus Bischoff, Karin Jacobs

**Affiliations:** † Experimental Physics, Center for Biophysics, Saarland University, Saarbrücken 66123, Germany; ‡ Department of Electrical Engineering, Trier University of Applied Science, Trier 54293, Germany; § Insitute of Medical Microbiology and Hygiene, 9379 Saarland University, Homburg 66421, Germany; ∥ VTT Technical Research Centre of Finland Ltd., Espoo 02150, Finland; ⊥ Medix Biochemica Group, Headquarter, Espoo 02180, Finland; # Max Planck School, Matter to Life, Heidelberg 69120, Germany

## Abstract

*Staphylococcus aureus* (*S. aureus*) is one of the bacterial species capable of forming multilayered biofilms on implants. Such biofilms formed on implanted medical devices often require the removal of the implant in order to avoid sepsis or, in the worst case, even the death of the patient. To address the problem of unwanted *S. aureus* biofilm formation, its first step, i.e., adhesion, must be understood and prevented. Thus, the development of adhesion-reducing surface coatings for implant materials is of utmost importance. In this work, we used single-cell force spectroscopy to analyze the adhesion of the biofilm-forming *S. aureus* strain SA113 on naive and protein-coated silicon surfaces (SiO_2_). In addition to the wild type, we used the SA113 Δ*dltA* knockout mutant to further investigate the effect of d-alanylation of lipoteichoic acids of the cell wall. In order to examine how the surface charge affects adhesion, we coated silanized SiO_2_ surfaces with amphiphilic class II hydrophobins. The naturally occurring hydrophobin HFBI was used as well as the HFBI variant D40Q/D43N, which is less negatively charged at physiological pH due to the exchange of two acidic aspartate residues. These two types of hydrophobin-coated surfaces resemble each other in roughness and wettability but differ only in charge. By measurement of the forces with which each *S. aureus* strain binds to hydrophobin-coated surfaces, we show that the adhesion of *S. aureus* at surfaces can be influenced by the charges exposed by the target surfaces. Therefore, in addition to hydrogen bonding, electrostatic interactions between the cell and the hydrophilic surface govern adhesion on these surfaces. Moreover, we found that for both HFBI coatings, the adhesion strength of *S. aureus* is reduced by nearly a factor of 30 compared to silanized SiO_2_ surfaces. Therefore, hydrophobin coatings are of great interest for further use in the field of biomedical surface coating.

## Graphical abstract

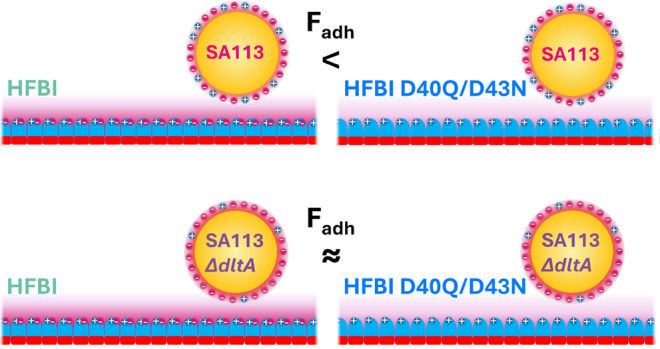

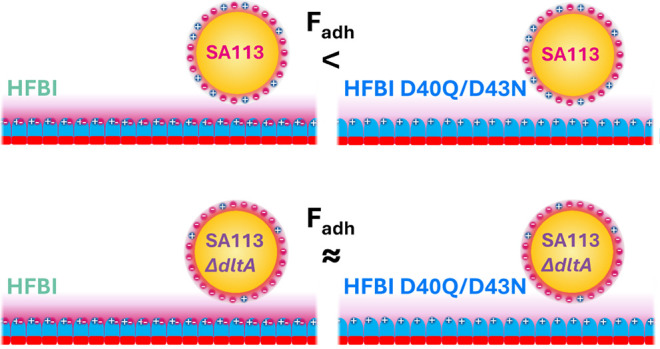

## Introduction

The ability of *Staphylococcus aureus* (*S. aureus*) to form biofilms on medical devices or to infect postsurgery wounds is well described.^1–4^ *S. aureus*-related catheter and bloodstream infections can dramatically increase patient morbidity,^5–8^ mortality,^9,10^ and healthcare costs.^11^ To address this burden, it is essential to gain a comprehensive understanding of the primary stages of biofilm formation,^12^ with a particular focus on the initial adhesion process. The state-of-the-art quantitative method for the investigation of bacterial adhesion is based on atomic force microscopy (AFM) and single-cell force spectroscopy (SCFS). SCFS allows quantifying adhesion forces, adhesion energies, and rupture lengths of single bacterial cells with the substrate.^13–20^

There are different strategies to prepare antifouling surfaces discussed in research such as, structured surfaces, natural antifoulings, zwitterionic coatings, etc.^21–23^ In addition to studying the adhesion forces on abiotic and biotic surfaces, modifying or changing the surface is also an excellent method to display antimicrobial properties.^24^ Antimicrobial effects of naturally occurring surfaces (silver and copper) have been described previously;^21^ however, in medical applications, it is not always possible to change the material of these devices, such as catheters, because they need to keep a certain flexibility while inserted into the patient to not harm them during movement. To achieve antimicrobial effects on such surfaces, a chemical coating or mechanical structuring is possible. Changing the surface roughness at the nanoscopic scale step by step has been shown to lower the adhesion^15^ while, at the same time, creating rifts in the size of bacterial cells increases the adhesion.^25,26^ Protein coatings are an alternative to structuring the surface for antimicrobial effects while keeping catheters biocompatible.^18^ The coating process with proteins reduces the adhesion, while simultaneously changing the surface charge or energy.^27–31^ Both characteristics are essential in *S. aureus* adhesion.^16,32^

In this paper, we investigate the effect of surface charge on the adhesion of *S. aureus* SA113 and the general impact of coating silicon with amphiphilic HFBI proteins produced by *Trichoderma reesei*.^33^ HFBI is a class II hydrophobin, creating stable interfacial protein monolayers,^34,35^ which can be used for surface coatings.^36,37^ In addition to the HFBI wild type, we use an HFBI variant, in which two aspartic acid amino acids are changed to glutamine and asparagine, to change the surface charge.^38^ Besides changing the charge of the coated surface, we also studied the impact of cell surface charge by employing a *S. aureus* mutant lacking d-alanylation of lipoteichoic acids (LTAs) on the cell wall (SA113 ΔdltA). To investigate the impact of cell surface charge, by employing a *S. aureus* mutant lacking d-alanylation of lipoteichoic acids (LTAs) on the cell wall (SA113 Δ*dltA*). This bacterial mutant allowed us to investigate the role of charge in cell-wall-associated adhesion factors during adhesion.

## Methods

### Hydrophobin Surface Coating

Class II hydrophobins, HFBI and HFBI D40Q/D43N, from the fungus *T. reesei* were prepared and purified at VTT (Espoo, Finland).^39^ The charge-based HFBI variant HFBI D40Q/D43N was initially prepared and characterized by Lienemann et al.^38^ Silane-coated (octadecyl-trichlorosilane, OTS)^40^ Si wafers (Siltronic AG, Burghausen, Germany) were used as hydrophobic adsorption substrates. The coating was done by adding a 60 μL drop of a 10 mM sodium acetate solution containing hydrophobins at a concentration of 4 μM to one OTS surface and placing a second surface on top. The setup was left for at least 30 min to allow the proteins to adsorb onto the OTS surface. The entire setup was next placed in deionized water to remove unbound proteins. The surfaces were then dipped several times into deionized water to remove any protein aggregates on top of the monolayer film. Protein films were imaged using an atomic force microscope (FastScan Icon, Bruker, Santa Barbara, CA, USA) to verify the complete protein coverage. Only the fully covered surfaces were afterward used for bacterial adhesion measurements. An optical contact angle meter (OCA25, DataPhysics Instruments GmbH, Filderstadt, Germany) with a direct dosing system (ESr-M) was used to determine the water contact angle (WCA) and phosphate-buffered saline (PBS) contact angle. Evaluation was performed using SCA20 dataphysics software.

### Human Serum Albumin Surface Coating

Similar to the HFBI coating, silicon surfaces were coated with human serum albumin (HSA, Merck Millipore, Darmstadt, Germany). Silane-coated (octadecyltrichlorosilane, OTS)^40^ Si wafers (Siltronic AG, Burghausen, Germany) were coated by adding a drop of HSA solution (4 mg in 1 mL of deionized water). The drop was left for 30 min before the surface was placed in a deionized water bath to remove nonabsorbed proteins. The surface was then carefully rinsed with deionized water to remove unbound aggregates. Protein coverage was measured using the same atomic force microscope as that described above for the HFBI coatings. Due to changes in the protein structure, measurements were performed in deionized water. Contact angle measurements were performed as described above for the HFBI-coated surfaces.

### Bacterial Strains and Growth Conditions

To study bacterial adhesion, the biofilm-positive *S. aureus* laboratory strain SA113 was utilized alongside the SA113 Δ*dltA* mutant,^41^ which was already used in a previous study.^16^ Both strains were provided by A. Peschel (University of Tübingen, Germany).^41,42^

The strains were grown on tryptic soy agar plates with 5% sheep blood (Becton Dickinson [BD], Heidelberg, Germany) and subsequently cultured in Tryptic Soy Broth (TSB, BD) in Erlenmeyer flasks at 37 °C and shaken at 150 rpm using a culture to flask volume of 1:10. The liquid cultures were inoculated the day before the experiment and incubated for 16 h. The next day, the overnight culture was diluted 100-fold to inoculate a new liquid culture, which was then grown for 2.5 h at 37 °C and 150 rpm to obtain exponential growth phase cells. 1 ml of this cell suspension was centrifuged for 3 min at 17,000*g*, and sedimented cells were washed twice with PBS (pH 7.4) to remove debris and extracellular material. Bacteria were then diluted 1:10 in PBS to prepare for SCFS.

### Bacterial Probes

Tipless cantilevers (MLCT-O10-D, Bruker-Nano, Santa Barbara, USA) were covered with a thin layer of polydopamine by polymerization of dopamine hydrochloride (99%, Sigma-Aldrich, St. Louis, MI, USA) in Tris buffer (pH 8.4). The cantilevers were dipped into the polydopamine solution for 1 h before being washed three times with water and dried under a flow bench. Next, a single bacterium was attached to a polydopamine-coated tipless AFM cantilever via a micromanipulator (Narishige Group, Tokyo, Japan). The preparation of the cantilevers and the immobilization of single bacterial cells were previously described by Thewes et al.^14^ Care was taken to ensure that the cells never dried out during probe preparation or force measurements. The cantilevers were calibrated before each measurement using the Sader method.^43^

### Single-Cell Force Spectroscopy (SCFS)

All force spectroscopy measurements with single bacterial probes were conducted under ambient conditions in PBS using a Nanowizard 4 instrument (Bruker Nano GmbH, Berlin, Germany). Force–distance curves were obtained using experimental parameter values previously determined in a study conducted by Spengler et al.:^16^ The ramp size was set to 800 nm, the force trigger (denoting the maximal force with which the cell is pressed onto the substrate) was 300 pN, and the retraction speed was 800 nm/s with a surface delay of 5 s. This time delay was chosen considering prior studies showing a correlation between cell adhesion strength and cell–surface contact time.^32,44–48^

Nine force–distance experiments with single, viable bacterial cells were performed on either an HFBI-, an HFBI-D40Q/D43N-, or an HSA-coated substrate. In total, 32 force–distance curves were recorded for each bacterial probe and substrate covering a 10 μm by 10 μm grid. Each individual cell/bacterium was tested on two of the three substrates, resulting in 64 force–distance curves per cell. Force–distance curves were analyzed using the JPKSPM Data Processing software, Version 7.0.128. An adhesion curve was defined as a nonadhesion event at adhesion forces below 40 pN, as this could not be distinguished from the noise of the baseline.

## Results and Discussion

### Characterization of HFBI Wild-Type, HFBI D40Q/D43N Coatings and HSA Coatings

To investigate the effect of surface charge on *S. aureus* adhesion, OTS-covered SiO_2_ surfaces were used, which were then coated with either wild-type HFBI or the HFBI D40Q/D43N variant. HSA-coated OTS surfaces were used to assess the influence of the HFBI coatings on adhesion. Due to the coating, the wettability of the HFBI (WCA: 25 ± 4°) and HFBI D40Q/D43N (WCA: 40 ± 2°) surfaces is greatly reduced compared to the OTS surface (WCA: 105 ± 3°), yet they have a higher WCA than the uncoated SiO_2_ surface (WCA: 7 ± 2°) (see Table 1, WCA). The strong amphiphilicity of HFBI molecules causes them to adsorb to the OTS with their hydrophobic side,^49^ exposing their hydrophilic side to the solution and rendering the surface hydrophilic. The HSA surfaces show an increased surface contact angle compared to the HFBI coatings (WCA: 81 ± 4°, see Table 1, WCA). As the contact angle measurements were carried out in air, this is probably due to the change in protein conformation^50^ and therefore incomplete surface coverage of the OTS by the blood plasma protein. Since all SCFS measurements were performed in PBS, the WCA was compared to the contact angle measured in PBS (Table 1, CA (PBS)). Overall, no major differences were observed between the contact angles in water and PBS. Minor variations can likely be attributed to the adsorption of salt ions present in the PBS buffer; however, these do not substantially affect the surface’s hydrophobicity.

**Table 1:** Surface Properties for the Surfaces Used in This Study, i.e., the Protein Coatings and the Uncovered Substrates Bare SiO_2_ and Silanized Silicon (OTS): WCA, PBS Contact Angle, Root-Mean-Square Roughness (RMSR), and Isoelectric Point (IEP)

	WCA/° (Water)	CA/° (PBS)	RMSR/nm	IEP
bare SiO_2_	7 ± 2	13 ± 2	0.14 ± 0.02 ^40^	<2 ^51^
OTS	105 ± 3	105 ± 3	0.17 ± 0.02 ^40^	≈ 3.0 ^51^
HFBI	25 ± 4	25 ± 5	0.33 ± 0.04	6.1 ^38^
HFBI D40Q/D43N	40 ± 2	34 ± 5	0.38 ± 0.07	7.0 ^38^
HSA	81 ± 4	85 ± 5	0.56 ± 0.1	4.8 ^52,53^

A slightly increased roughness is measured on the HFBI-, HFBI D40Q/D43N-, and HSA-coated surfaces (HFBI: 0.33 nm, HFBI D40Q/D43N: 0.38 nm, and HSA: 0.56 nm), but the samples still have a root-mean-square roughness (RMSR) well below 1 nm (see Table 1, RMSR). The OTS is homogeneously covered by the hydrophobin coating and shows no structural differences between the HFBI and HFBI D40Q/D43N coatings in the range of bacterial size (see Figure 1), so no major impact on bacterial adhesion due to the roughness is expected.^15^ However, as reported earlier by Lienemann et al.,^38^ HFBI and HFBI D40Q/D43N have a crucial difference: They differ in their IEP (Table 1, IEP). While the IEP of HFBI is at pH 6.1, the IEP of HFBI D40Q/D43N is at pH 7.0. Therefore, coating OTS with HFBI and HFBI D40Q/D43N provides surfaces with similar wettability, roughness, and chemistry but differences in charge.

**Figure 1:**
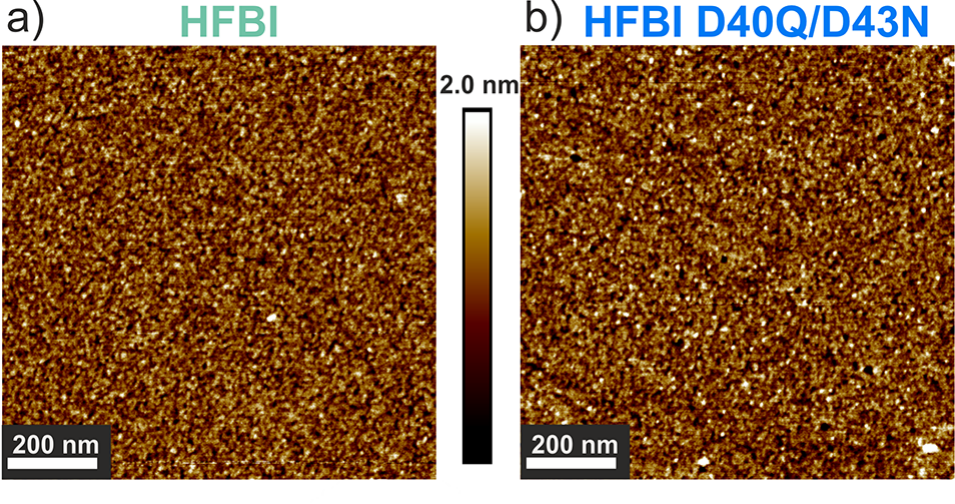
Images were captured using the off-resonance tapping mode PeakForce Tapping (Bruker). AFM images (1 μm^2^, 512 × 512 pixel) of HFBI (a) and HFBI D40Q/D43N (b) coatings.

### Comparing the Adhesion on Bare SiO_2_ to OTS-, HFBI-Coated Surfaces

It has already been reported that protein surface coatings influence the adhesion of *S. aureus* to the implant material.^18,54,55^ For example, initial measurements of bacteria revealed reduced bacterial adhesion on hydrophobin-coated surfaces.^56,57^ Furthermore, effects on the adsorption of a second layer of proteins on a hydrophobin coating have also been reported.^38,58,59^ It has been suggested that electrostatic interactions may play a key role in this adsorption.^58^

To evaluate the detailed effect of HFBI coatings on *S. aureus* adhesion, adhesion was compared on silicon, silane-coated silicon (OTS), and on HFBI-coated OTS surfaces. Adhesion measurements on SiO_2_ and OTS have been previously described by Maikranz et al.^32^ in detail, demonstrating that *S. aureus* exhibits significantly stronger adhesion to the hydrophobic OTS surface compared to the hydrophilic SiO_2_, with median adhesion forces of 22.4 nN and 1.0 nN, respectively. In the present study, the adhesion of *S. aureus* to hydrophilic, protein-based surface coatings was found to be even weaker than to SiO_2_ (Figure 2). Specifically, the median adhesion force on SiO_2_ remained in the nanonewton (nN) range (1.0 nN), while the median adhesion force on HFBI surfaces was reduced to 156.0 pN. The box-and-whisker plots of Figure 2 contain all measured adhesion forces on these surfaces, including the nonadhesion events (adhesion force < 40 pN). The absolute magnitude of these adhesion forces is relevant for understanding the underlying biological interactions. Adhesion forces in the nN range suggest the involvement of many tethered macromolecules—consistent with strong, cumulative binding on hydrophobic surfaces—whereas piconewton-scale forces, as observed on HFBI, indicate only a few weak or nonspecific interactions.

**Figure 2:**
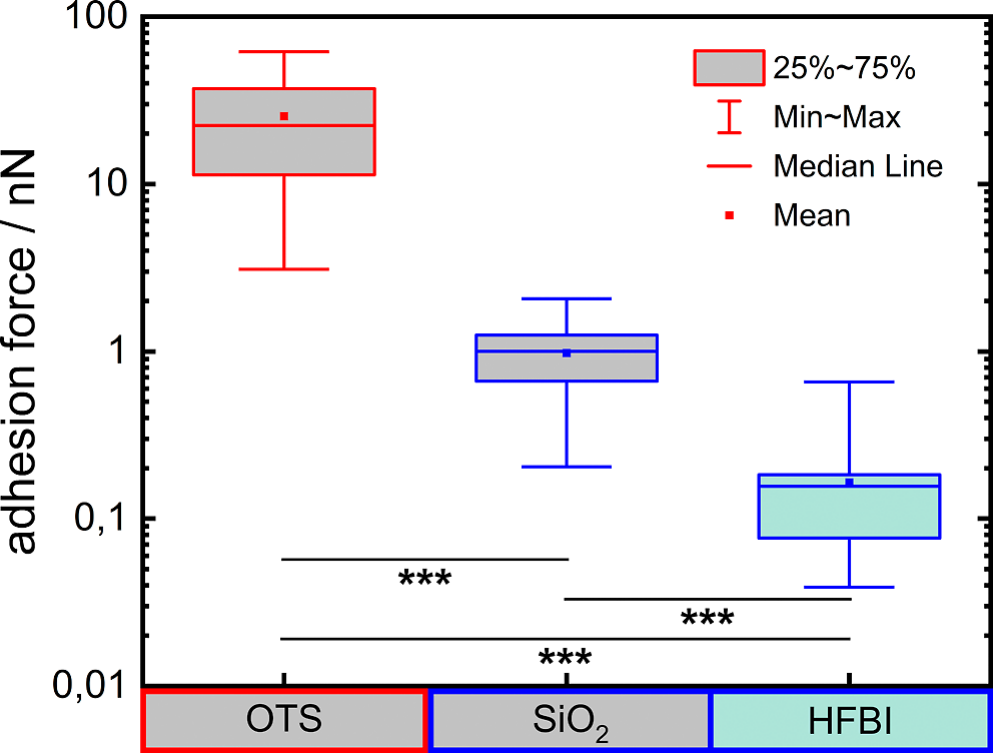
Min-to-max box plots of the adhesion force of *S. aureus* on OTS, SiO_2_,^32^ and HFBI surfaces. Displaying the median adhesion forces (OTS: 22.4 nN, SiO_2_: 1.0 nN, and HFBI: 156.0 pN) and the mean adhesion forces (OTS: 25.4 nN, SiO_2_: 977.3 pN, and HFBI: 164.3 pN) on these three different surfaces. The border color of the box plots indicates the degree of hydrophobicity of the surface (red: hydrophobic and blue: hydrophilic). The adhesion forces on all three surfaces differ significantly (*p* < 0.001, Mann–Whitney-*U* test).

The increased adhesion on the OTS surfaces can be explained by the wettability of the surface (Table 1, WCA). The shift in the adhesion force of *S. aureus* on OTS (WCA: 105 ± 3°) and SiO_2_ (WCA: 7 ± 2°) surfaces was described in previous studies^13,32,60^ and could be explained by the number and strength of tethering macromolecules to the surface.^32^ While many macromolecules can adhere weakly to the hydrophobic surfaces, only a few strong binding macromolecules can attach to the hydrophilic surfaces. Therefore, the difference between adhesion on the OTS- and HFBI-coated surfaces (WCA: 25 ± 4°) can also be described by the effect of surface wettability. However, when comparing the adhesion on HFBI-coated surfaces with the adhesion on SiO_2_ surfaces, it becomes clear that the wettability of the surfaces cannot serve as the sole explanation for the differences, as the measured WCA is higher on HFBI-coated surfaces (see Table 1, WCA). Furthermore, as it has previously been shown that RMSR values much greater than 10 nm are required to reduce *S. aureus* adhesion,^15^ the slightly higher roughness of the protein-coated surfaces compared to SiO_2_ is not likely to be responsible for the difference in adhesion. A possible explanation for the reduced adhesion on HFBI-coated surfaces compared to SiO_2_ is the diminished surface area for the formation of hydrogen bonds. Coating the surface with HFBI protein creates a more chemically heterogeneous surface that may reduce the binding ability of the bacterial macromolecules.

### Influence of the Surface Charge on the Adhesion of *S. aureus*

To study the effect of the electrostatic interactions on the adhesion of *S. aureus*, SCFS measurements with immobilized cells on HFBI-coated surfaces were performed. Besides HFBI, the HFBI variant D40Q/D43N with an altered surface charge pattern was employed (Table 1, IEP).^38^ In addition to *S. aureus* SA113, SA113 Δ*dltA* was used as a control. The Δ*dltA* mutant lacks the gene *dltA* encoding the d-alanine-d-alanyl carrier protein ligase. DltA catalyzes the first step in the d-alanylation of LTAs. Consequently, this mutant strain’s cell wall and LTAs lack d-alanine, resulting in an increased negative surface charge of the cell wall.^16,61^

SCFS data recorded with SA113 cells on a HFBI D40Q/D43N-coated surfaceare presented here, normalized to the mean force of the same cell determined on a HFBI-coated surface (Figure 3a). This normalization guarantees a direct comparison of the change in adhesion on the HFBI compared to the HFBI D40Q/D43N surface, whereby the heterogeneity among the cells, caused, e.g., by cell wall heterogeneity, patchiness, and age, of the bacterial cells is ruled out. An increase in the normalized mean adhesion force of SA113 on the HFBI D40Q/D43N surfaces is evident from a comparison of the SCFS data with those recorded on the more negatively charged HFBI surfaces (lower IEP). On average, the adhesion force is twice as high on HFBI D40Q/D43N than on HFBI, with the adhesion force of individual cells ranging from 50 to 330%. Notably, in this series of experiments, 7 out of 9 SA113 cells tested displayed increased adhesion to the HFBI D40Q/D43N surface. Moreover, the probability of nonadhesion events is drastically reduced on HFBI D40Q/D43N compared to HFBI: Over all measurements, only about 10% were classified as nonadhesion on HFBI D40Q/D43N, compared to about 40% on HFBI. In the histograms in Figure 3 these nonadhesion events are shown separately (zero value). This also shows a clear shift in the adhesion force distribution toward higher values for the HFBI D40Q/D43N variant (Figure 3b). The distribution of all nonzero adhesion events of SA113 cells is not significant (*p* = 0.28, unpaired *t*-test). The box-and-whisker plots in Figure 3 underline the shift of the adhesion force distribution, showing an even greater shift because the nonadhesion events are included here.

**Figure 3:**
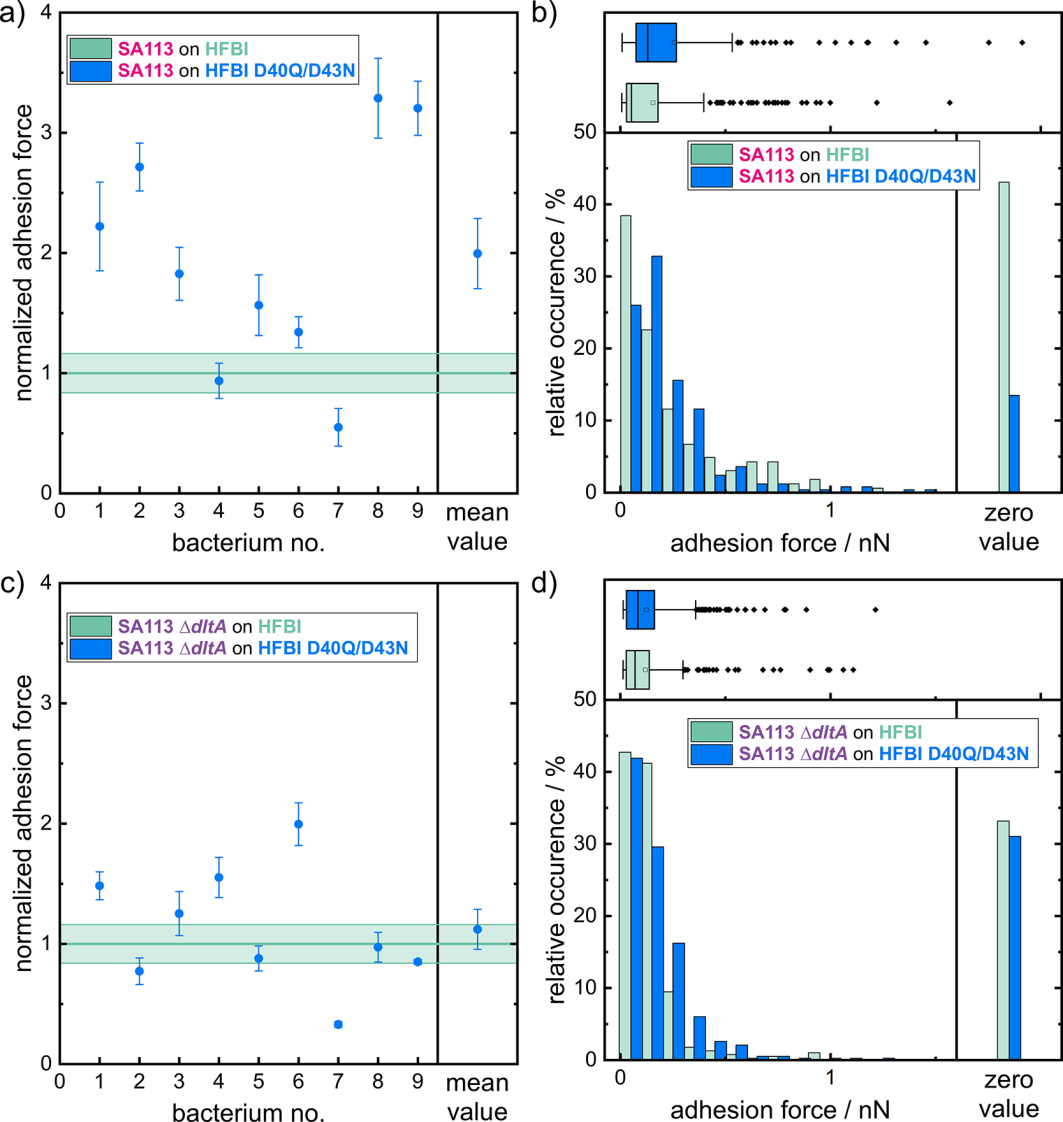
Adhesion forces of nine *S. aureus* SA113 (a,b) and SA113 Δ*dltA* (c,d) cells on hydrophobin surfaces with different IEPs. (a,c) Mean adhesion forces of single *S. aureus* cells normalized to the adhesion value of each cell on the HFBI surfaces (HFBI: pale green line and HFBI D40Q/D43N: blue measurement points). Error bars depict the standard error of the mean. (b,d) Histograms and box-and-whisker plots of all adhesion force values measured for *S. aureus* cells. Nonadhesion events (adhesion force <40 pN) are excluded from the main histogram and are shown separately (right panel), but are included in the box-and-whisker plots (upper panel). (b,d) The box-and-whisker plots display the mean adhesion forces of SA113 (HFBI: 155.1 pN and HFBI D40Q/D43N 256.0 pN) and the median adhesion forces (HFBI: 53.0 pN and HFBI D40Q/D43N 130.0 pN), as well as the mean adhesion forces of SA113 Δ*dltA* (HFBI: 118.7 pN and HFBI D40Q/D43N 123.8 pN) and median adhesion forces (HFBI: 69.8 pN and HFBI D40Q/D43N 83.5 pN).

Performing the same series of measurements with SA113 Δ*dltA* cells on HFBI and HFBI D40Q/D43N revealed an overall decrease in adhesion forces compared to SA113 (Figure 3d) and is consistent with similar trials performed on SiO_2_ surfaces in an earlier study.^16^ Also unlike SA113, the majority of cells of the Δ*dltA* mutant adhered to surfaces covered with HFBI D40Q/D43N with a reduced force (5 out of 9) when compared to HFBI-covered surfaces (Figure 3c). However, the normalization of the adhesion forces did not show a clear trend between the adhesion force on HFBI and HFBI D40Q/D43N, with a normalized mean for the adhesion force on HFBI D40Q/D43N of 1.1 compared with the adhesion force on HFBI (Figure 3c). The histogram of all adhesion forces measured with the SA113 mutant Δ*dltA* supports the statement of a low impact of the surface to the adhesion of SA113 Δ*dltA*. Nonadhesion measurements are in the same order of magnitude (30–35%) on both HFBI surfaces. A minimal shift of the measurable adhesion forces to higher values was seen on the HFBI D40Q/D43N surfaces, but this was marginal (Figure 3d) and also evident in the box and whisker plots above the histogram. The adhesion of all SA113 Δ*dltA* cells measured with an adhesion event showed no significant difference in adhesion forces between the two surfaces tested (*p* = 0.84, unpaired *t*-test).

Our measurements performed with strain SA113 demonstrate clearly an influence of electrostatic interactions on the adhesion of *S. aureus* to hydrophilic surfaces: A reduced number of negative charges at the surface of the protein coating leads not only to a measurable increase in adhesion force but also to an increased probability for adhesion.

In contrast, the reasons for the indistinguishable adhesion of the *S. aureus* strain SA113 Δ*dltA* to surfaces coated with either HFBI or HFBI D40Q/D43N in terms of adhesion force and nonadhesion frequency remained unclear. However, the adhesion strength of the negatively charged Δ*dltA* strain to both types of HFBI protein coating was lower than the adhesion of the SA113 wild-type strain (see Figure 3b,d), suggesting an impact of the cellular surface charge of the *S. aureus* cell for adhesion to the tested surfaces. The assumption that the higher surface charge of the cell wall is the direct cause for this decrease is, however, contrasted by the observation that no difference in the binding strength of the Δ*dltA* mutant to the zwitterionic HFBI and the monopolar HFBI D40Q/D43N surface could be resolved. However, it might also be reasonable to assume that the electrostatic attraction between the anionic Δ*dltA* mutant and the positive charges on the hydrophobin surfaces dominates the adhesion process. If this is the case, then the negative HFBI wild-type charges might not be detectable, thus explaining the results in Figure 3c. Another aspect may be more important: The effect of deletion of the *dltA* gene on the composition of the bacterial cell wall is not yet fully understood. Previous work has shown that the lack of DltA leads to a lower autolysin activity^62,63^ because the highly charged teichoic acids are involved in the control of Atl activity. Atl is a major cell wall hydrolase in *S. aureus* and, therefore, an autolysin.^64^ The reduced autolysin activity might result in a different cell wall composition^65,66^ and most likely influences the patchiness of the distribution of the cell-wall-associated proteins.^65^ These patches of adhesins are vital for strong adhesion, as shown by Spengler et al. using an SCFS approach combined with simulations.^17^ Therefore, to understand the exact effects of such knockout mutants, more detailed investigations of the indirect changes of the macromolecules in the cell wall are indispensable.

In addition, we compared the adhesion of SA113 and SA113 Δ*dltA* on HFBI-coated surfaces with HSA-coated surfaces by normalizing SCFS data to the mean adhesion force determined on HFBI. We chose HSA as a comparison because it is a commonly used protein for coating surfaces in medical research.^67,68^ The normalized mean adhesion force of all individual bacterial cells of each strain is displayed in the Supporting Information in Figure S1. The mean adhesion force of SA113 Δ*dltA* (2.1 ± 0.2 nN) on HSA-coated surfaces compared to SA113 (0.8 ± 0.1 nN) is doubled. This increase is particularly surprising, given that the adhesion of SA113 Δ*dltA* was almost the same on HFBI and HFBI D40Q/D43N, while SA113 showed a twofold increase in adhesion force on the latter coating. Even more unexpected is the increase in the adhesion force of SA113 Δ*dltA* on HSA, given that on OTS-coated and bare SiO_2_ surfaces, the adhesion of SA113 Δ*dltA* was consistently lower than that of SA113.^16^ The most plausible explanation is the almost complete denaturation of HSA on OTS.^50^ This could result in a charge distribution that is particularly favorable for SA113 Δ*dltA*. Since the exact composition of the cell surface of SA113 Δ*dltA* and its displayed components or its patchiness is still unclear, this could, in combination with the denaturation of HSA, lead to positive interactions between HSA on OTS and SA113 Δ*dltA*, hence an increase in adhesion force. The denaturation is also a significant weak point of HSA compared to HFBI. HFBI-coated surfaces are stable in air and liquid, while HSA changes its displayed conformation with a change of the medium. The high controllability of the surface and its properties make HFBI-coated surfaces preferable to the HSA-coated ones when studying the effects of surfaces on bacterial adhesion.

## Conclusions

SCFS measurements of bacterial adhesion demonstrate distinct behaviors on HFBI-coated and bare SiO_2_ surfaces, showing a clear reduction in adhesion on HFBI-coated surfaces compared to that on untreated silicon. Different HFBI variants therefore offer a promising alternative to established protein coatings such as HSA for reducing bacterial adhesion. In the case of hydrophilic surfaces, bacterial adhesion is largely driven by electrostatic interactions between the surface and the macromolecules of the bacterial cell wall. For instance, the adhesion of *S. aureus* on hydrophilic HFBI-coated surfaces is predominantly influenced by these electrostatic forces. The composition and arrangement of cell wall macromolecules can further modify the hierarchy of the binding forces involved. For example, with the *S. aureus* Δ*dltA* mutant strain, the influence of surface charge is substantially reduced, resulting in lower adhesion overall. This could be linked to the reduced autolysin activity in Δ*dltA* cells, which likely alters the distribution of adhesion molecules across the bacterial cell wall. The patchy distribution of adhesins appears to be advantageous, enabling bacteria to adhere more effectively, despite such changes. Comparing hydrophobic OTS surfaces with hydrophilic HFBI-coated OTS surfaces, a significant reduction in bacterial adhesion becomes evident. This reduction highlights the role of additional factors, such as surface wettability and hydrogen bonding capacity. Typically, bacterial adhesion is enhanced on hydrophobic surfaces due to stronger hydrophobic interactions, but HFBI disrupts this tendency, demonstrating a remarkable ability to lower adhesion on such surfaces.

The experimental results of this study are thus in line with established findings regarding (i) the influence of surface hydrophobicity^32^ and (ii) the role of bacterial cell surface charge^16^ on the adhesion behavior of *S. aureus*. They demonstrate how protein coatings can be utilized to partially control bacterial adhesion due to the change in hydrophobicity and charge of the available surface. Beyond that, exploiting the exceptional stability of HFBI coatings and site-specific mutations of these molecules enabled precise control of charges at the protein film and thus yielded detailed insight into their influence on the adhesion of *S. aureus*. In particular, the observation that adhesion forces on the mutated hydrophobin HFBI D40Q/D43N were, on average, twice as high as on unmodified HFBI highlights the relevance of surface charge in modulating bacterial attachment. Since the experimental framework employed in this study is not restricted to *S. aureus*, its application to a broader spectrum of bacterial species may yield critical insights into species-specific adhesion phenomena, thereby enriching our fundamental understanding of microbial surface interactions and informing the rational design of next-generation antiadhesive materials. In future research, the development of HFBI coatings into antibacterial coatings holds great biomedical potential. Hydrophobin fusion proteins could be designed not only to prevent bacterial adhesion but also to kill attached bacteria. Enzymatically active hydrophobin fusion proteins have already been produced, offering the possibility of coating surfaces with these proteins.^69,70^ Additionally, a deeper understanding of the patchy distribution of bacterial cell wall macromolecules and its influence on adhesion forces would be critical for advancing our knowledge of bacterial adhesion mechanisms.

## Supplementary Material



## Notes

Adhesion forces of nine *S. aureus* SA113 and SA113 Δ*dltA* cells on HFBI- or HSA-coated surfaces (PDF)
