# Supplementary material for: Staphylococcus aureus Adhesion on Hydrophobin Coatings: Adhesion Forces and the Influence of Surface Charge
Source: ACS Omega. 2025 Aug 20;10(34):38376–84. doi: 10.1021/acsomega.4c11010 (PMC12409555; doi:10.1021/acsomega.4c11010)
Supplement: Supplementary file 1 [file ao4c11010_si_001.pdf]

Staphylococcus aureus adhesion on hydrophobin coatings: adhesion forces and the influence of surface charge

Friederike Nolle,[†,‡,#] Ben Wieland,[¶,#] Kirstin Kochems,[†] Hannah Heintz,[†] Michael Lienemann,[§,‖] Philipp Jung,[¶] Hendrik Hähl,[†] Markus Bischoff,[¶] and Karin Jacobs[*,†,⊥]

†*Experimental Physics, Saarland University, Center for Biophysics, 66123 Saarbrücken, Germany*
‡*Department of Electrical Engineering, Trier University of Applied Science, 54293 Trier, Germany*
¶*Insitute of Medical Microbiology and Hygiene, Saarland University, 66421 Homburg, Germany*
§*VTT Technical Research Centre of Finland Ltd., 02150 Espoo, Finland*
‖*Medix Biochemica Group, Headquarter, 02180 Espoo, Finland*
⊥*Max Planck School, Matter to Life, 69120 Heidelberg, Germany*
#*These authors contributed equally to this work.*

E-mail: k.jacobs@physik.uni-saarland.de

Supporting Information

The bacterial strains were grown and prepared for SCFS, and the surfaces were coated as described in S1. SCFS data recorded with SA113 or SA113 *ΔdltA* cells on an HSA-coated surface were analyzed by normalizing to the mean force of the same cell determined on an HFBI-coated surface (Fig. S1a or S1c). Histograms and box-and-whisker plots show the distribution of the recorded adhesion forces, either with non-adhesion events included in box-and-whisker plots or separated in the histograms (Fig. S1b or S1d).

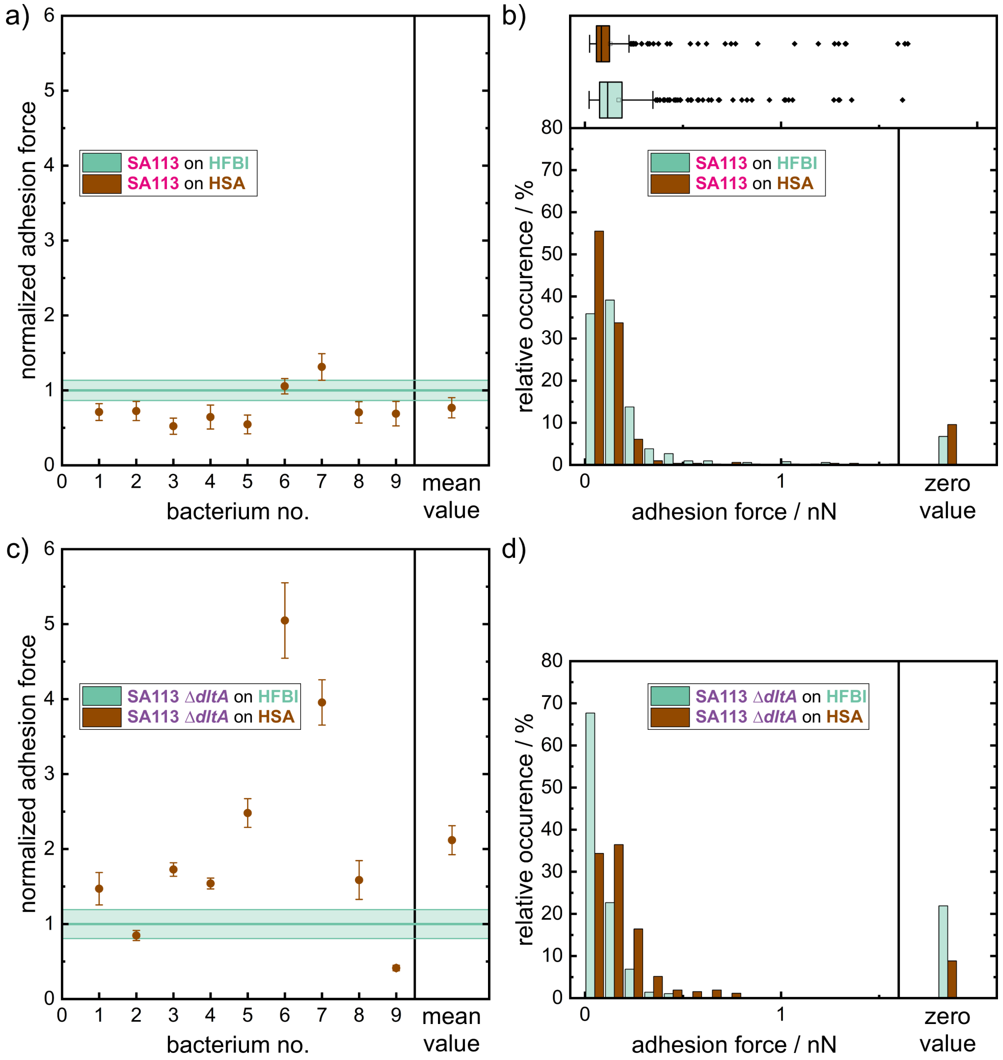

Figure S1: Adhesion forces of nine *S. aureus* SA113 (a,b) and SA113 *ΔdltA* (c,d) cells on HFBI or HSA coated surfaces. (a,c) Mean adhesion forces of single *S. aureus* cells normalized to the adhesion value of each cell on the HFBI surfaces (HFBI: pale green line, HSA: brown measurement points). Error bars depict the standard error of the mean. (b,d) Histograms and box-and-whisker plots of all adhesion force values measured for *S. aureus* cells. Display also the non-adhesion events (adhesion force $< 40\,\text{pN}$) excluded from the main histogram, included in the box-and-whisker plots. (b,d) The box-and-whisker plots display the mean adhesion forces of SA113 (HFBI: 172.8 pN, HSA 129.5 pN) and the median adhesion forces (HFBI: 116.1 pN, HSA 84.1 pN), as well as the mean adhesion forces of SA113 *ΔdltA* (HFBI: 97.8 pN, HSA 182.1 pN) and median adhesion forces (HFBI: 69.0 pN, HSA 139.3 pN).
